# Mechanisms Underlying the Regulation of Innate and Adaptive Immunity by Vitamin D

**DOI:** 10.3390/nu7105392

**Published:** 2015-09-24

**Authors:** Ran Wei, Sylvia Christakos

**Affiliations:** Department of Microbiology, Biochemistry and Molecular Genetics, New Jersey Medical School, Rutgers, the State University of New Jersey, 185 South Orange Ave, Newark, NJ 07103, USA; weira@njms.rutgers.edu

**Keywords:** vitamin D, cathelicidin, autoimmune disease

## Abstract

Non-classical actions of vitamin D were first suggested over 30 years ago when receptors for the active form of vitamin D, 1,25-dihydroxyvitamin D_3_ (1,25(OH)_2_D_3_), were detected in various tissues and cells that are not associated with the regulation of calcium homeostasis, including activated human inflammatory cells. The question that remained was the biological significance of the presence of vitamin D receptors in the different tissues and cells and, with regard to the immune system, whether or not vitamin D plays a role in the normal immune response and in modifying immune mediated diseases. In this article findings indicating that vitamin D is a key factor regulating both innate and adaptive immunity are reviewed with a focus on the molecular mechanisms involved. In addition, the physiological significance of vitamin D action, as suggested by *in vivo* studies in mouse models is discussed. Together, the findings indicate the importance of 1,25(OH)_2_D_3_ as a regulator of key components of the immune system. An understanding of the mechanisms involved will lead to potential therapeutic applications for the treatment of immune mediated diseases.

## 1. Introduction

Although the importance of vitamin D for curing rickets has been known for over 80 years, in the past several years there has been a renewed interest in vitamin D. This is due not only to an increased awareness of the importance of vitamin D for mineralization but also to the realization that vitamin D has many other effects including preventing or, at least partially, protecting against a number of autoimmune diseases (for example, experimental autoimmune encephalomyelitis (EAE; the murine model of multiple sclerosis, MS) and inflammatory bowel disease (IBD). Vitamin D has also been reported to regulate innate immunity by increased production of antimicrobial peptides and subsequent killing of bacteria. In spite of the potential importance of vitamin D over a wide range of immune disorders, the mechanisms involved in vitamin D regulation of immune responses and how vitamin D is involved in linking innate and adaptive immunity have not been clearly defined and are topics of ongoing investigation. 1,25(OH)_2_D_3_, the hormonally active form of vitamin D, is produced by two sequential hydroxylations: by 25-hydroxylase in the liver (it has been suggested that CYP2R1, which is also present in extra-hepatic sites, is the key vitamin D 25-hydroxylase in the liver) and by 25-hydroxyvitamin D 1α hydroxylase (CYP27B1) in the kidney [1,2,3]. The actions of 1,25(OH)_2_D_3_ are mediated, similar to other hormones, by a nuclear receptor (vitamin D receptor, VDR) which heterodimerizes with the retinoid X receptor and binds to vitamin D response elements (VDREs) in target genes and modulates their transcription [4,5,6]. 1,25(OH)_2_D_3_ modulates two separate, but interacting, types of immunity: innate and adaptive. Effects on each system, including molecular mechanisms involved, will be reviewed and *in vivo* studies in mouse models will be discussed.

## 2. Effects of Vitamin D on Innate Immunity

**Figure 1:**
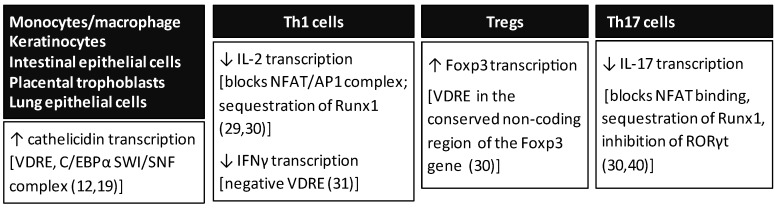
1,25(OH)_2_D_3_ is a key transcriptional regulator of components of the immune system. Summary of transcriptional mechanisms by which 1,25(OH)_2_D_3_ modulates both innate and adaptive immunity.

As a first line of defense against infection the innate immune system is responsible for responding rapidly and recognizing and eliminating invading pathogens to prevent exacerbation of infection. The innate immune system involves activation of Toll-like receptors (TLRs; pathogen recognition receptors) in monocytes leading to the induction of antimicrobial peptides including cathelicidins, and the subsequent killing of bacteria. Cathelicidins are a family of proteins that originate from a precursor molecule. The *C*-terminal 37 amino acid peptide, which is responsible for broad antimicrobial action, is activated by proteolysis from the pro-peptide [7]. The only known human cathelicidin, hCAP18 (the *C* terminal domain of human cationic antimicrobial protein 18 or LL-37), was first identified in 1995 [8]. It is encoded by the human cathelicidin antimicrobial peptide (CAMP) gene. Although first identified in neutrophils, CAMP is also expressed in monocytes, dendritic cells, lymphocytes, natural killer (NK) cells, and epithelial cells of the skin, respiratory tract, and gastrointestinal tract. CAMP has broad antibacterial activity against both Gram positive and Gram negative bacteria [9]. Mechanisms of CAMP antimicrobial action include attraction of the cationic CAMP to the bacterial membrane due to interaction with the anionic surface components of the bacterial membrane. The accumulation of CAMP induces a curvature strain in the lipid membrane bilayer and translocation of CAMP from the outer membrane to the surface of the inner membrane resulting in disruption of bacterial membrane homeostasis [10]. 1,25(OH)_2_D_3_ has been reported to be a major regulator of CAMP not only in monocytes but also in lung and intestinal epithelial cells, keratinocytes, and trophoblasts of the placenta [11,12,13,14,15,16]. In monocytes it has been reported that activation of TLR2/1 in combination with 1,25(OH)_2_D_3_ stimulates the expression of CAMP which is correlated to an enhancement of monocyte mediated killing of *Mycobacterium tuberculosis* [17]. In keratinocytes 1,25(OH)_2_D_3_ increases TLR2/1 and CAMP expression, resulting in increased antimicrobial activity against *Staphylococcus aureus* [15,18]. 1,25(OH)_2_D_3_ as an autocrine/paracrine regulator of immunity during pregnancy is suggested by the 1,25(OH)_2_D_3_ induction of CAMP in placental trophoblasts (which is independent of the TLR signaling pathway) [16]. Induction of CAMP in lung epithelial cells by 1,25(OH)_2_D_3_ (which also correlates with increased antibacterial activity) is also independent of TLR signaling [13]. Recent studies have shown that C/EBPα is a potent enhancer of CAMP transcription in lung epithelial cells and that C/EBPα functionally cooperates with VDR and Brm (a component of the SWI/SNF chromatin remodeling complex) in regulating CAMP transcription [19] (Figure 1). In light of the increased prevalence of antibiotic resistant pathogens, these findings, which define novel mechanisms involved in the regulation of CAMP, suggest potential candidates for increasing innate immunity to infection that would not depend on antibiotic administration. Further studies related to the regulation of CAMP have shown that histone acetylation can enhance 1,25(OH)_2_D_3_ regulation of CAMP in different cell types [18,20]. The use of histone deacetylase inhibitors is an additional novel approach of strengthening innate immunity to treat bacterial infections [21]. In addition to CAMP, 1,25(OH)_2_D_3_ mediated VDR action has also been reported to converge with the TLR induced interleukin 1 beta (IL-1β) signaling pathway to induce the expression of the antimicrobial peptide defensin beta 4 (DEFB4; formally HBD2) in monocytes [22]. Additional mechanisms by which vitamin D induces innate antimicrobial effector responses include induction of reactive oxygen intermediates and activation of antibacterial autophagy [23,24]. Although these *in vitro* findings present convincing evidence of 1,25(OH)_2_D_3_-mediated antimicrobial activity, further studies are needed to determine the effect of 1,25(OH)_2_D_3_ *in vivo* on host resistance to bacteria. Since 1,25(OH)_2_D_3_ regulation of CAMP is specific to humans and non-human primates [12], future studies using a transgenic humanized mouse expressing the human CAMP gene (generated by the A. Gombart lab, personal communication) will provide important insight into mechanisms and effects of 1,25(OH)_2_D_3_ *in vivo* in response to infection.

## 3. Vitamin D and the Adaptive Immune Response

Although 1,25(OH)_2_D_3_ promotes the innate immune response, it is a suppressor of adaptive immunity [25,26,27]. 1,25(OH)_2_D_3_ suppresses immune responses mediated by Type 1 T helper (Th1) cells which are capable of producing inflammatory cytokines. Inflammatory cytokines produced by Th1 cells and repressed by 1,25(OH)_2_D_3_ include IL-2 and interferon gamma (IFNγ) [28]. A direct effect of 1,25(OH)_2_D_3_/VDR on IL-2 and IFNγ transcription has been reported. The suppressive effect of 1,25(OH)_2_D_3_ on IL-2 transcription results, at least in part, from the blocking of NFAT/AP-1 complex formation by VDR/retinoid X receptor (RXR) and association of VDR/RXR with the NFAT element in the IL-2 promoter as well as sequestration of Runx1 by 1,25(OH)_2_D_3_/VDR [29,30] (Figure 1). With regard to IFNγ transcription, repression by 1,25(OH)_2_D_3_ has been shown to involve direct binding of VDR/RXR to negative vitamin D responsive elements in the IFNγ promoter [31] (Figure 1). In contrast to the effect on Th1 cytokines, 1,25(OH)_2_D_3_ has been reported to enhance cytokines associated with Th2 cells [32,33]. It has been suggested that the 1,25(OH)_2_D_3_ mediation of the Th2 response, which can result in the suppression of Th1 cell responses, may be indirect, may depend on the interaction of multiple cell types and the outcome of the regulation may be determined by the physiological condition [32,34]. 1,25(OH)_2_D_3_ also induces regulatory T (T_reg_) cells (cells that are important for the inhibition of inflammation) as indicted by 1,25(OH)_2_D_3_-mediated induction of Foxp3 (the transcription factor involved in the development and function of T_reg_ cells) [30,35,36,37]. 1,25(OH)_2_D_3_ has been shown to up-regulate Foxp3 at the transcriptional level (a VDRE was identified in the highly conserved non-coding region of the mouse *Foxp3* gene) [30] (Figure 1). In addition to Th1, Th2, and Treg cells (subsets from the CD4 + T cell lineage), IL-17 producing T cells are a distinct subset termed Th17 cells. IL-17, which has been implicated in the pathogenesis of a number of autoimmune diseases, is inhibited by 1,25(OH)_2_D_3_ [30,38,39]. The mechanism of the negative effect of 1,25(OH)_2_D_3_ on IL-17 involves blocking NFAT and Runx1 binding to the IL-17 promoter and induction of Foxp3 (which binds to and inhibits NFAT and Runx1) [30]. 1,25(OH)_2_D_3_ may also result in suppression of IL-17 through inhibition of the transcription factor RORγt which is required for the differentiation of Th17 cells [40] (Figure 1). 1,25(OH)_2_D_3_ also inhibits differentiation of dendritic cells resulting in a suppression of the pro-inflammatory cytokine IL-12 and an increase in IL-10, an anti-inflammatory cytokine produced by Th2 cells [41,42,43]. In addition to blocking dendritic cell differentiation, IL-12 production is also repressed by 1,25(OH)_2_D_3_ at the level of transcription (VDR/RXR has been reported to bind to the NF-κB site in the IL-12p20 promoter, thus preventing activation by NF-κB) [44]. It is increasingly recognized that actions of 1,25(OH)_2_D_3_, similar to other steroids, are complex, involving regulation of gene activity at a range of locations, often many kilobases from the gene’s start site. Although the mechanisms involved in VDR gene regulation in immune cells are beginning to be addressed, further genome-wide studies are needed to define the multiple control regions and global networks involved in 1,25(OH)_2_D_3_ regulation of immune function.

## 4. Vitamin D and Autoimmune Diseases (MS and IBD)

**Figure 2:**
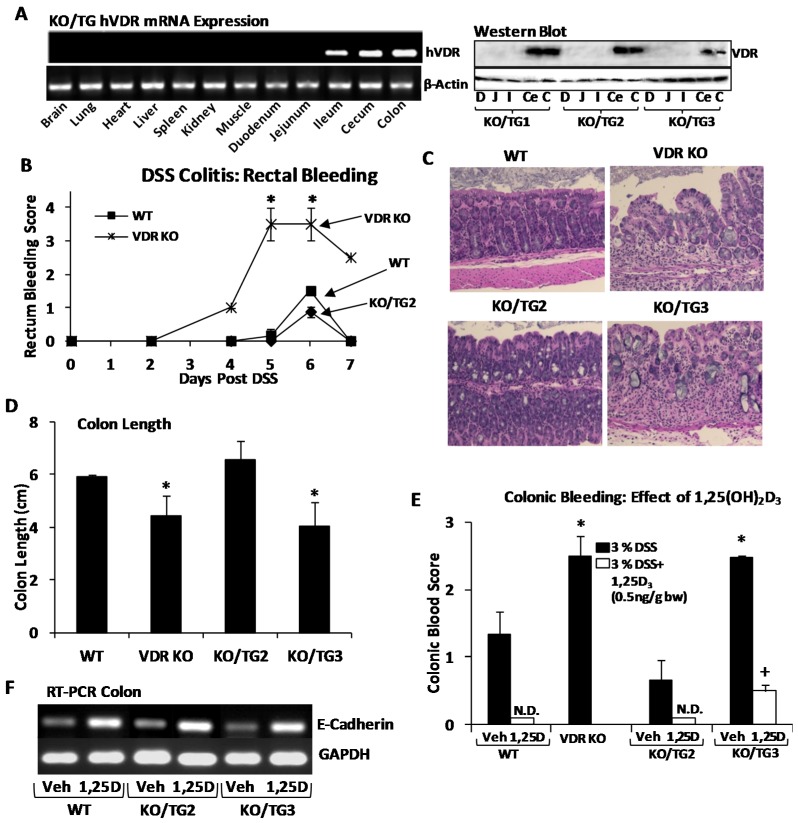
Transgenic mice (Knockout/transgenic 2 (KO/TG2)) expressing vitamin D receptor (VDR) exclusively in the distal intestine at levels equivalent to wild type (WT) are less sensitive to colitis induced by dextran sulfate sodium (DSS) than VDR KO mice. Symptoms of colitis are more severe in the KO/TG3 line which expresses low levels of VDR exclusively in the distal intestine. Colonic bleeding is attenuated in in both KO/TG2 and KO/TG3 mice by 1,25(OH)_2_D_3_ treatment. (**A**) Left panel: Real time-PCR (RT-PCR) hVDR mRNA: VDR KO mice expressing hVDR specifically in ileum, cecum, and colon were generated (results shown are for KO/TG2: for all three KO/TG lines VDR mRNA was only observed in ileum, cecum and colon); Right panel: Representative Western blot of VDR using 50 µg of nuclear protein (D, duodenum; J, jejunum; I, Ileum; Ce, cecum; C, colon). Ponceau S staining was used as the loading control. (**B**) Rectal bleeding score following DSS administration. Mice consumed 3% DSS *ad libitum* in the drinking water for six days and resumed water alone for one day before killing. Rectal bleeding was assessed on a scale from 0 to 4. Since male and female mice responded similarly to DSS, both genders were used in the DSS colitis experiments. (**C**) Representative H & E stained colon sections from mice killed on day seven post initiation of DSS treatment. Severe crypt disruption and infiltration of immune cells are observed in VDR KO and KO/TG3 mice. (**D**) Colonic length of mice killed seven days post initiation of the DSS treatment; (**E**) Colonic blood score (0, no blood detected to 3, blood visible throughout the colon) of mice killed on day seven post initiation of DSS treatment. No blood was detected (N.D.) in WT or KO/TG2 mice treated with 1,25(OH)_2_D_3_ (0.5 ng/g bw, ip) one day prior to DSS administration and every other day thereafter. 1,25(OH)_2_D_3_ treatment of KO/TG3 mice resulted in an attenuated blood score. Veh; vehicle (propylene glycol). * *p* < 0.05 compared to WT and KO/TG2. + *p* < 0.05 compared to vehicle treated KO/TG3 mice. Values are means ± SEMs, *n* = 3–4 per group. (**F**) Representative RT-PCR analysis of E-cadherin mRNA expression in colon of DSS treated WT, KO/TG2 and KO/TG3 mice receiving vehicle (Veh) or 1,25(OH)_2_D_3_. RT-PCR is representative of 3 separate experiments for WT and KO/TG2 mice and two separate experiments for KO/TG3 mice. All experimental procedures were approved by the Institutional Animal Care and Use Committee of Rutgers, New Jersey Medical School.

The geographic incidence of MS and IBD indicates a negative correlation between increased sun exposure and MS and IBD prevalence [45,46,47]. MS and IBD are almost unknown in equatorial regions. Since the synthesis of vitamin D in the skin from 7 dehydrocholesterol by ultraviolet (UV) irradiation is the most important source of vitamin D (few foods contain vitamin D) and vitamin D deficiency has been associated with increased incidence of MS and IBD [48,49], a beneficial effect of vitamin D for prevention and treatment of these autoimmune diseases has been suggested. It should be noted that the negative correlation between sun exposure and MS may be due, in part, to vitamin D but may also be due to the previously reported effects of UV irradiation on the immune system independent of vitamin production [50,51]. In the study of Becklund *et al.* [51] EAE (the mouse model of MS) was repressed by UV irradiation even when serum calcium and 25(OH)D_3_ levels were not elevated. 1,25(OH)_2_D_3_, when given prior to and post immunization, prevents the induction of EAE (maintenance of the animals on a normal or high calcium diet is required for the inhibitory effect of 1,25(OH)_2_D_3_) [52,53,54]. 1,25(OH)_2_D_3_ treatment of mice with ongoing EAE results in the reversal of paralysis [30,53]. 1,25(OH)_2_D_3_ inhibition of EAE is associated with inhibition of IL-17 and IL-12 and induction of IL-10 and Treg cells [30,55,56,57]. Although 1,25(OH)_2_D_3_ inhibits EAE and VDR is necessary for this inhibition, studies in VDR knockout (KO) mice have shown that EAE is less severe in VDR null mice and the onset is delayed, suggesting that VDR is needed for the development of immune responses involved in the induction of EAE [58,59]. With regard to IBD, VDR KO mice develop more severe symptoms of IBD which are accompanied by increased numbers of IL-17 and IFNγ secreting cells [60]. Treatment with 1,25(OH)_2_D_3_ of experimentally-induced colitis in WT mice reduces inflammation [57,60]. Although suppression of Th1 and Th17 cells and induction of Th2 and T_reg_ cells have been reported to be responsible for the beneficial effect of 1,25(OH)_2_D_3_ in IBD, induction of DSS (dextran sulfate sodium) colitis in T cell deficient mice has been shown, suggesting that other pathways are also involved in the protective effect of 1,25(OH)_2_D_3_ in experimental IBD [61]. Recently mice expressing VDR exclusively in the distal ileum and in the cecum and colon of VDR KO mice (KO/transgenic (TG) mice) were generated and used as a model to determine the role of intestinal epithelial cells in the susceptibility of VDR KO mice to IBD (Figure 2; [62,63]). The full length human (h)VDR cDNA under the control of the 9.5 kb Cdx2 promoter (from E. Fearon; [64]) which directs transgene expression specifically in the ileum, cecum and colon was used to obtain founders that resulted in high (TG1 and 2) or low (TG3) hVDR expression. TG mice were mated to VDR KO mice to obtain mice with VDR expressed exclusively in the distal intestine (Figure 2A). In the KO/TG1 and 2 mice, which have a similar phenotype, transgenic expression of VDR restricted to the distal intestine rescued the skeletal abnormalities observed in the VDR KO mice and serum parathyroid hormone (PTH) and calcium levels were normalized [62,65]. In the KO/TG3 mice (low VDR expression; less than 50% of the levels of VDR protein observed in KO/TG1 and 2 mice, Figure 2A, Western blot) serum calcium levels were similar to VDR KO mice and PTH levels remained elevated but were significantly less than the levels observed in the VDR KO mice [62]. The skeletal abnormalities in VDR KO mice were not rescued in KO/TG3 mice but were not as severe as in the VDR KO mice [62]. KO/TG2 and KO/TG3 mice were used for DSS colitis studies (Figure 2B–F). KO/TG2 mice, which express VDR only in the epithelial cells of the distal intestine at levels equivalent to wild type (WT) mice, were more resistant to DSS colitis when compared to VDR KO mice (displaying a milder form of colitis, similar to WT mice, including marked reductions in rectal bleeding and colonic blood score; Figure 2B–E: KO/TG2). Also, at seven days post DSS treatment weight loss was observed in VDR KO mice (88% ± 3% of the original weight) but not in WT or KO/TG2 mice. In the transgenic line KO/TG3, with low VDR expression, DSS treatment resulted in more severe symptoms (Figure 2C–E: KO/TG3). 1,25(OH)_2_D_3_ treatment improved symptoms of DSS colitis in these mice as well as in the WT and KO/TG2 mice (Figure 2E open bar), suggesting that even low levels of VDR in intestinal epithelial cells can respond to 1,25(OH)_2_D_3_ treatment to reduce symptoms of IBD. 1,25(OH)_2_D_3_ treatment was associated with induction of E-cadherin mRNA in the distal intestine (Figure 2F). Since VDR is absent from immune cells in the KO/TG mice but present in the epithelial cells of the distal intestine, these findings suggest a critical role for intestinal epithelial cells in the maintenance of epithelial cell integrity and suppression of IBD by 1,25(OH)_2_D_3_/VDR. Since increased calcium intake has previously been reported to ameliorate the symptoms of IBD in transgenic rats by strengthening the mucosa barrier [66], it is possible that an increase in calcium, mediated by 1,25(OH)_2_D_3_ treatment, may also play a role in the suppression of IBD by 1,25(OH)_2_D_3_.

Additional mechanisms suggested to be involved in the protective effect of 1,25(OH)_2_D_3_ in IBD include regulation of the composition of the gastrointestinal microflora and a reduction in intestinal epithelial cell apoptosis [67,68]. Administration of antimicrobial peptides has been proposed as a therapeutic strategy against IBD [69]. Thus, it is possible that effects on antimicrobial peptides are also involved in maintenance of intestinal homeostasis by 1,25(OH)_2_D_3_ and are not restricted to 1,25(OH)_2_D_3_ effects related to infectious diseases.

## 5. Conclusions

1,25(OH)_2_D_3_ has potent effects on both innate and adaptive immune responses. Although, unlike vitamin D deficiency and rickets, a causal link between vitamin D deficiency and immune disorders has not yet been proven, there is compelling evidence in the laboratory of beneficial effects of 1,25(OH)_2_D_3_ in inflammatory diseases where Th1 cytokines have a pathological role. It is possible that induction of antimicrobial peptides as well as suppression of adaptive immunity may be involved in the protective effect of 1,25(OH)_2_D_3_ in certain autoimmune diseases. Since inflammatory cytokines can have a protective role against infection, the physiological consequences of modulation of the immune system by 1,25(OH)_2_D_3_ are complex and may not always be beneficial. Further *in vivo* studies examining the relationship between vitamin D and host resistance to infection are needed.
